# Development of a New Marker System for Identification of* Spirodela polyrhiza* and* Landoltia punctata*

**DOI:** 10.1155/2017/5196763

**Published:** 2017-01-12

**Authors:** Bo Feng, Yang Fang, Zhibin Xu, Chao Xiang, Chunhong Zhou, Fei Jiang, Tao Wang, Hai Zhao

**Affiliations:** ^1^Chengdu Institute of Biology, Chinese Academy of Sciences, Chengdu 610041, China; ^2^Chengdu University, Chengdu 610106, China

## Abstract

Lemnaceae (commonly called duckweed) is an aquatic plant ideal for quantitative analysis in plant sciences. Several species of this family represent the smallest and fastest growing flowering plants. Different ecotypes of the same species vary in their biochemical and physiological properties. Thus, selecting of desirable ecotypes of a species is very important. Here, we developed a simple and rapid molecular identification system for* Spirodela polyrhiza* and* Landoltia punctata* based on the sequence polymorphism. First, several pairs of primers were designed and three markers were selected as good for identification. After PCR amplification, DNA fragments (the combination of three PCR products) in different duckweeds were detected using capillary electrophoresis. The high-resolution capillary electrophoresis displayed high identity to the sequencing results. The combination of the PCR products containing several DNA fragments highly improved the identification frequency. These results indicate that this method is not only good for interspecies identification but also ideal for intraspecies distinguishing. Meanwhile, 11 haplotypes were found in both the* S. polyrhiza* and* L. punctata* ecotypes. The results suggest that this marker system is useful for large-scale identification of duckweed and for the screening of desirable ecotypes to improve the diverse usage in duckweed utilization.

## 1. Introduction

The Lemnaceae (duckweed) comprise a widespread family of monocotyledonous plants growing in water [1–3]. It is an ideal plant with several unique properties such as fast reproduction through gemmation and high protein content and it can absorb large amounts of nutrients such as nitrogen (N) and phosphorus (P) [4–6]. As a result, duckweed has shown great potential in recovering nutrients from wastewater.

*Spirodela polyrhiza* and* Landoltia punctata* are two species of Lemnaceae which are widely distributed around the world. On the morphological basis, the identification of these two species (*L. punctata* also is a genus) is commonly based on the number of roots. Normally,* S. polyrhiza* has seven to 21 roots with one root (rarely two) perforating the prophyllum.* L. punctata* has two to seven roots per frond; however, one and up to 12 roots were rarely observed. Sometimes, a spot is found in* S. polyrhiza* because of the accumulation of anthocyanin under limitation of nutrients. Fronds of* L. punctata* often have a red dorsal surface.

*S. polyrhiza* is considered as a potential energy crop which can be used for bioethanol production due to its fast growth rate and starch accumulation capability. The protein content of some ecotypes of* S. polyrhiza* grown on anaerobically treated swine wastewater was found to reach as high as 45% of the dry weight [7, 8]. With the removal of nutrients, the starch content could be increased by 59.3% within four days at 5°C [9]. After the fermentation of the enzymatic hydrolysis of the duckweed biomass, the ethanol content was corresponding to 50.9% of the original dry duckweed biomass [8].

*S. polyrhiza* and* L. punctata* can be used as a stable and efficient gene expression system [10]. The rapid clonal growth and simple axenic culture have made them suitable laboratory subjects for researching such diverse topics as photoperiod, leaf morphogenesis, and toxicology on plants [11]. Aprotinin, a small serine protease inhibitor used in human health, has been stably transformed and expressed in* Spirodela* [12].

Being extremely reduced in morphology, miniaturization of organs, and its wide distribution, as well as high phenotypic plasticity in response to environmental conditions, have made taxonomy of Lemnaceae a great challenge [3, 13]. As a result, the employment of genetic markers for identification of duckweed at the inter- and intraspecies-level is used to confirm the morphological classification results [14–16]. Previously, genetic markers such as RAPD, AFLP, and DNA barcode were employed for identification of the phylogenetic relationship of duckweed [2, 17]. The DNA barcode markers based on cp-DNA sequences were for species identification [18]. The marker atpF-atpH noncoding spacer was considered to be able to serve as a universal marker for species-level identification.

However, no marker was reported to be useful for ecotype (intraspecies) level identification so far. Most significant diagnostic value is at specie level. For example, the number of roots:* L. punctata* normally possesses two to seven roots per frond. Seven to 21 roots are present in* S. polyrhiza* (Martirosyan et al. 2009). Different ecotypes of the same species vary significantly in their biochemical and physiological properties. Under the low temperature, some ecotypes show several times higher turion formation capacity than other ecotypes from* S. polyrhiza*. By using 27 ecotypes, the range of number of turions formed per frond was ranged from 0.22 to 5.9 (Kuehdorf et al. 2014). Under standardised cultivation conditions, the relative growth rate and weekly yields of 12 ecotypes from* L. punctata* were determined. Relative growth rate ranged from 0.253 to 0.509 days (−1) and relative yields from 5.9 to 17.8 weeks (−1). Meanwhile, the result shows that relative growth rate does not vary primarily at the level of the species but rather reflects the adaptation of individual ecotypes to specific condition (Ziegler et al. 2014). Under the same treatment, the starch content of the different ecotypes from the same species varies significantly. For* L. punctata*, the starch content of ecotypes ZH1010 and ZH1031 [19] was 27.3% and 18.18%, respectively. For* S. polyrhiza*, the starch content of ecotypes ZH1045 and ZH1027 was 14.9% and 30.1%, respectively. Meanwhile, the component and amount of the flavonoids with the same condition vary greatly (unpublished result, data not shown).

In this study, we present the development of new markers for intraspecies identification of duckweed. We establish a simple and accessible protocol to construct a database against the individual duckweed which could be validated. Many new ecotypes were found by using this method.

## 2. Materials and Methods

### 2.1. Plant Materials

A worldwide collection of duckweed has been characterized by morphologic classification. Duckweed was classified according to Les et al. [14]. From this collection, 48 ecotypes of* L. punctata* and 49 ecotypes of* S. polyrhiza *were selected. 18 ecotypes were collected from the Institut für Integrative Biologie (Zürich, Switzerland). The ecotypes used encompass the worldwide geographic distribution ranging from 5 m to 1890 in altitude (Table 1; Table  S1, in Supplementary Material available online at https://doi.org/10.1155/2017/5196763). After collection, these ecotypes were maintained in LB plates. A summary of all ecotypes included in this study was listed in Table  S1.

### 2.2. Analysis Using DNA Barcoding Markers

Previously, three noncoding spacers (atpF-atpH, psbK-psbI, and trnH-psbA) were used for genetic analysis of duckweed [18]. To validate the efficiency of the three markers, the PCR amplification and products sequencing were conducted. All the ecotypes of* L. punctata* and* S. polyrhiza* were used. Total DNA was extracted using CTAB. The primers sequences and PCR amplification condition were conducted according to Wang et al. [18]. The PCR products were fractionated using 2% agarose gels. The fragments were cloned into pGEM-T vector and sequenced by automatic DNA sequencing. Each product was sequenced at least three times.

### 2.3. Development and Validation of SSR Marker

48 pairs of primer designed by the sequence of the genomic and chloroplast DNA were synthesized by Invitrogen Company. For validation of the primers, two ecotypes of duckweed from* Landoltia* and* Spirodela *were used as template. The primers showing good ability to detect the polymorphism among the accessions were selected for further analysis (Table 2).

PCR was carried on the Mastercycler Thermal Cycler (Eppendorf). For amplification, a total of 50 *μ*L reaction contained 50 ng of genomic DNA, 25 *μ*L of 2x Buffer, 0.5 mM of each of the dNTPs, 0.25 mM of MgCl_2_, 0.5 *μ*M of forward and reverse primers, and 2 units of KOD Plus Polymerase (TOYOBO). The PCR conditions were one cycle of 95°C for four min and 28 cycles of 94°C for 30 sec, 52°C for 30 sec, and 72°C for 30 sec, followed by a final extension of 72°C for 10 min.

### 2.4. Analysis of PCR Products Using the Applied Biosystems 3730 DNA Analyzer

PCR amplification by using the selected primers was conducted and the products were diluted 1 : 30 in water. One *μ*L of the diluted PCR products was added to 7 *μ*L of the HIDI-formamide and 0.1 *μ*L of GeneScan 500 LIZ size standard (Applied Biosystems, Forster City, CA). The mixtures underwent denaturation at 95°C for 10 minutes and then were analyzed in the Applied Biosystems 3730 DNA Analyzer. The patterns of the DNA fragment were analyzed with GeneMarker V2.2.0 software.

## 3. Results

### 3.1. Length Polymorphisms among Different Duckweeds by DNA Barcoding Markers

Previously, three DNA barcoding markers (atpF-atpH, psbK-psbI, and trnH-psbA) were detected to be easy for amplification and good for identification of different type of duckweeds. In this study, 97 ecotypes were used (48* L. punctata* and 49* S. polyrhiza*) and the PCR products length was detected. The results show only one type of length was found for primers atpF-atpH (683 bp) and trnH-psbA (273 bp) in* S. polyrhiza*. psbK-psbI acquired two types of products length (501 bp and 531 bp) in* S. polyrhiza*. For* L. punctata*, one type of product length was found in atpF-atpH (662 bp) and psbK-psbI (522 bp) primers PCR reaction. Three types of products length (489 bp, 484 bp, and 273 bp) were detected in trnH-psbA primer PCR reaction. All the three pairs of primers show high success for PCR amplification, with 100% success for atpF-atpH and psbK-psbI and 98% success for trnH-psbA (Table 3).

### 3.2. Validation of Markers Designed

For identification of different ecotypes, the primers designed should meet two basic requirements: first is high success rate of PCR amplification and the second is high PCR products polymorphism to permit different ecotypes to be distinguished. To evaluate these 48 primers, genomic DNA extracted from ecotypes (12* S. polyrhiza* and 12* L. punctata*) was used for PCR amplification. To meet the two requirements, three markers were found suitable (both high PCR amplification success and high polymorphism) for all ecotypes identification.

**Figure 1:**
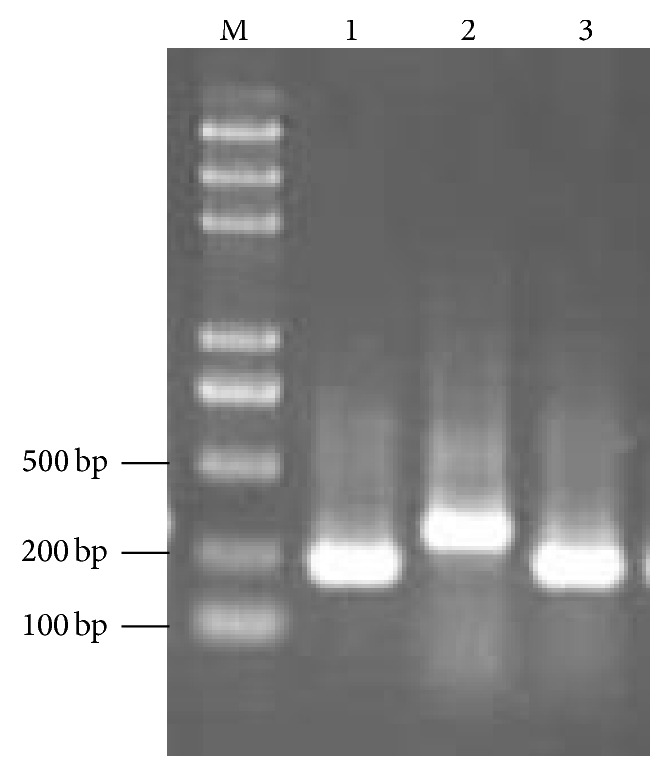
Electrophoresis of the PCR products amplified from duckweed (*Landoltia punctata* ecotype ZH0001-S-0) with the designed three pairs of primers in an agarose gel. M: DNA ladder Marker III (100, 200, 500, 750, 1,000, 2,000, 3,000, and 5,000 bp; Tiangen Biotech Co., Ltd.). Line 1: primers SC19/20. Line 2: primers SC35/36. Line 3: primers SC09/10.

For* L. punctata*, the PCR product length of primers SC09/10, SC19/20, and SC35/36 was quite variable, with 186–256 bp, 222–284 bp, and 234–298 bp, respectively (Figure 1; Table 2). All the three pairs of primers show high success for PCR amplification, with 95% success for SC09/10, 90% success for SC19/20, and 100% success for SC35/36. Meanwhile, some ecotypes gave more than one fragment (mostly two fragments). For* S. polyrhiza*, the PCR product length of primers SC09/10, SC19/20, and SC35/36 also showed significant variability, with 170–254 bp, 212–266 bp, and 224–298 bp, respectively (Figure 1; Table 2). All the three pairs of primers show high success for PCR amplification, with 92% success for SC09/10, 95% success for SC19/20, and 100% success for SC35/36. Two fragments were detected in some ecotypes.

### 3.3. Length Polymorphism of Duckweeds Revealed by Applied Biosystems 3730 DNA Analyzer

To efficiently identify the polymorphism of each ecotype, the PCR products of three pairs of primers were mixed and then measured by Applied Biosystems 3730 DNA Analyzer. As a result, each ecotype product mixture contained fragments from three pairs of primers. This made the detection more efficient and cost lower. Theoretically, at least three fragments could be found from each ecotype except for some unsuccessful PCR amplification which resulted in two fragments. 11 haplotypes were detected in* Landoltia* and* Spirodela* ecotypes, and most of them contained three fragments. Two and three haplotypes were found with only two fragments in* Landoltia* and* Spirodela* ecotypes, respectively. Four and three haplotypes were detected with four fragments in* Landoltia* and* Spirodela*, respectively. Only one haplotype was comprised of five fragments in both species (Table 3).

## 4. Discussion

Duckweed has been researched intensively for its promising usage in bioenergy, biomedicine, and waste water treatment. A* Spirodela polyrhiza* (160 Mb) has been selected for whole genome sequencing by DOE-JGI community sequencing program. With genome sequence information, the gene discovery and functional verification could be conducted in this aquatic monocot family. Meanwhile, from a systematic view, the morphological character combined with the DNA sequence method could resolve the classification problem. However, until the sequencing of many ecotypes of duckweed, the identification of this family could be conducted by genetic markers. Indeed, many genetic markers have been designed and used for the phylogenetic study [18, 20].

In this study, we validated the most useful DNA barcoding markers for duckweed previously reported and designed new markers for ecotype identification [18]. Ecotypes from two species of* S. polyrhiza* and* L. punctata* were selected for analysis. These ecotypes represent a worldwide collection which resulted highly accessible for phylogenetic and genomic research (Table 1). Meanwhile, ecotypes from these two species are easy to be collected and morphological classified.

First, the DNA barcoding markers were found useful for interspecies identification but not suitable for intraspecies distinguishing. Previously, the DNA barcoding markers atpF-atpH, psbK-psbI, and trnH-psbA were selected for duckweed species identification [18]. To validate these three markers for ecotypes identification, PCR amplification and products sequencing were conducted as reported. High success of PCR amplification was acquired with only three PCR amplifications failed (Table 3). For DNA barcoding marker atpF-atpH, 48 ecotypes of* L. punctata* and 49 ecotypes of* S. polyrhiza *were found both with only one length of PCR products. As a result, this marker was not suitable for discriminating the different ecotype from these two species because none of the polymorphism was detected (Table 3). The similar results were found in DNA barcoding marker psbK-psbI. Two length polymorphisms were found of the marker psbK-psbI for ecotypes from* L. punctata *and three length polymorphisms were found of the marker trnH-psbA for ecotypes from* L. punctata *(Table 3). As a result, these three markers were good for species identification not suitable for ecotype discrimination.

**Figure 2:**
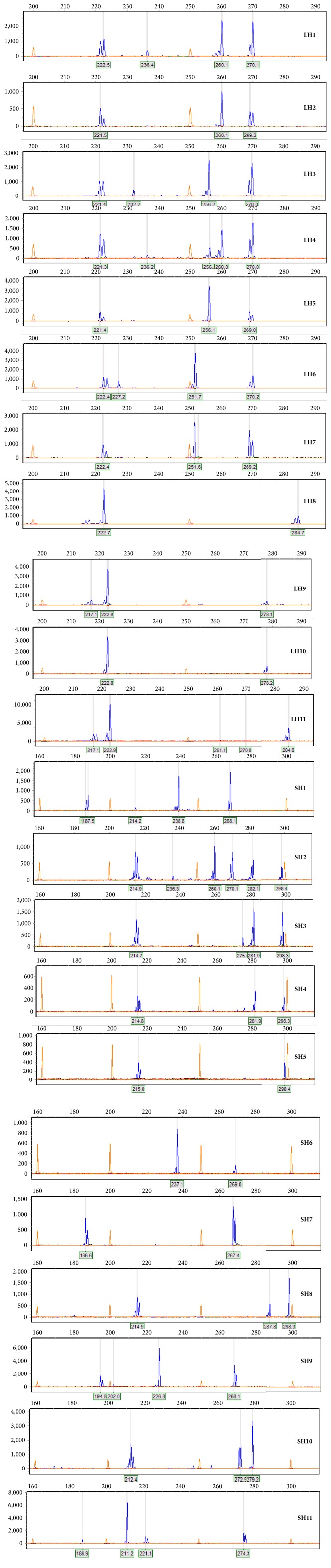
Electropherograms showing capillary electrophoresis separation of the PCR product fragments amplified from ecotypes with the three primers. The horizontal axis displays the size of the detected PCR product fragments, while the vertical axis presents the intensity of the signal (i.e., the indicator of concentration of fragments in the PCR products). The orange peaks match the standard fragments in the GeneScan 500 LIZ size standard, while the blue ones represent the PCR products fragments amplified from different ecotypes. The numbers on the horizontal axis represent the size of the corresponding peak in the GeneScan 500 LIZ size standard (orange). “LH” represents* Landoltia punctata* haplotype number and “SH” represents* Spirodela polyrhiza* haplotype number. Different haplotypes displayed different types of blue peaks (DNA fragments) and combinations.

Because of the low polymorphism detected from DNA barcoding markers, we designed new markers for intraspecies identification of duckweed. 48 pairs of primers were designed and validated by two types of ecotypes. According to two criterions (the high success of PCR amplification and polymorphism between the two ecotypes), three markers were found. The length of PCR products was highly polymorphic for all these three markers, with a range of 170–254 bp, 212–266 bp, and 224–298 bp, respectively (Table 2; Figure 2). Furthermore, for detecting of more polymorphisms, the products of three markers were mixed and fractionated by Applied Biosystems 3730 DNA Analyzer which is a capillary electrophoresis. This electrophoresis can discriminate one base pair of the PCR products with several fragments. This method is highly effective and accurate and costs much less than sequencing for polymorphism detection (Figure 2).

By using this method, 11 haplotypes of* S. polyrhiza* and 11 haplotypes of* L. punctata* were found in 97 ecotypes (Table 4). On average, four ecotypes can find one haplotype, which is much efficient compared to DNA barcoding markers used. As a result, more and more haplotypes could be found and discriminated. The geographic differentiation showed significant influence on the genetic haplotypes in our study. For instance, haplotype 7 was found in Australia and pacific area in ecotypes of* S. polyrhiza. *Meanwhile, several haplotypes were detected worldwide. Haplotype 2 was in ecotypes of* L. punctata* found around the world with high presence. These two findings suggested that both the geographic differentiation and conservation were the feature of duckweed.

In conclusion, we designed new markers which could serve as a universal marker for inter- or intraspecies-level identification of* S. polyrhiza *and* L. punctata*. These markers combined with the capillary electrophoresis will significantly lower the cost and improve the efficiency of duckweed distinguishing especially at ecotype level. Thus, many new ecotypes with different physiological properties could be screened. Therefore, this new marker system is a significant contribution to the identification of duckweed.

## Supplementary Material

97 ecotypes of duckweed were collected and most of them were from China. The ecotypes from Zhao's lab were recorded its location, GPS information and altitude. Meanwhile, the haplotypes of these ecotypes were also presented.

## Figures and Tables

**Table 1:** Number of ecotypes per country and geographical area for samples characterized.

Country	*Landoltia*	*Spirodela*
	Sample number	Sample number
China	39	23
Vietnam	3	8
South America	1	2
Asia	1	6
Europe	1	9
Australia	2	0
Pacific	1	0
Africa	0	1

**Table 2:** Three pairs of primers designed and used well for duckweed ecotype identification. “*T *(°C)” represents the degenerate temperature.

Primers	Primer sequences (5′-3′)	*T* (°C)	PCR product length range
SC09/10	TTAGTATTGTGCGACATTCG	52	170–256 bp
	TTTCTTTGATTTGAACTCCC		
SC19/20	GCGTTCTGTTTCTTTACCTA	53	212–284 bp
	CGGAGTAGAGCAGTTTGG		
SC35/36	ACCCTGGAGCATACCTTG	53	224–298 bp
	AGGATTAGGAATGGGCGT		

**Table 3:** Success ratios of PCR amplification and sequencing results of three pairs of designed markers.

	atpF-atpH		psbK-psbI		trnH-psbA	
	*Spirodela*	*Landoltia*	*Spirodela*	*Landoltia*	*Spirodela*	*Landoltia*
Length of product 1	662	683	522	531	489	273
Length of product 2	—	—	—	501	484	—
Length of product 3	—	—	—	—	273	—
% success of PCR	100%	100%	100%	100%	100%	98%

**Table 4:** Multilocus haplotypes defined with three pairs of primers. Alleles codes correspond to the size of the PCR products.

*Landoltia* haplotype number	SC09/10, SC19/20, SC35/36
1	221, 236, 260, 270
2	221, 260, 269
3	221, 232, 256, 270
4	221, 236, 256, 260, 270
5	222, 256, 270
6	222, 227, 251, 270
7	222, 252, 270
8	222, 284
9	217, 223, 278
10	222, 278
11	217, 223, 261, 285

*Spirodela* haplotype number	SC09/10, SC19/20, SC35/36

1	187, 214, 238, 268
2	214, 260, 270, 282, 298
3	214, 275, 282, 298
4	214, 282, 298
5	214, 298
6	237, 268
7	186, 267
8	214, 287, 298
9	195, 226, 268
10	212, 272, 279
11	187, 211, 221, 274
