# Supplementary material for: Development of a New Marker System for Identification of Spirodela polyrhiza and Landoltia punctata
Source: Int J Genomics. 2017 Jan 12;2017:5196763. doi: 10.1155/2017/5196763 (PMC5266846; doi:10.1155/2017/5196763)
Supplement: Supplementary file 1 — 97 ecotypes of duckweed were collected and most of them were from China. The ecotypes from Zhao's lab were recorded its location, GPS information and altitude. Meanwhile, the haplotypes of these ecotypes were also presented. [file 5196763.f1.docx]

**Table S1**

| specie | Accession number | Accession name | Location | GPS | Altitude (m) | Haplotype number |
| --- | --- | --- | --- | --- | --- | --- |
| *Landoltia*  *punctata* | ZH0001-S-0 | S3 | Chengdu, China | 104/30 | 498 | 1 |
|  | ZH0005-S-0 | 1# | Chengdu, China | 104/30 | 496 | 5 |
|  | ZH0011-S-0 | V5 | Huzhimin,  Vitnam | 106/10 | 5 | 2 |
|  | ZH0035-S-0 | J2 | Chengdu, China | 104/30 | 486 | 3 |
|  | ZH0175-S-3 | YN11-2 | Yunnan, China | 102/24 | 876 | 3 |
|  | ZH0044-S-5 | Q4-2 | Qionglai, China | 103/30 | 455 | 4 |
|  | ZH0057-S-5 | Y3-1 | Yaan,China | 103/30 | 576 | 3 |
|  | ZH0069-S-5 | Y8-1 | Yaan,China | 103/30 | 909 | 9 |
|  | ZH0076-S-5 | Y11-2 | Yaan,China | 103/29 | 646 | 1 |
|  | ZH0115-S-8 | Q8-1 | Qionglai, China | 103/30 | 637 | 9 |
|  | ZH0118-S-8 | Q10-1 | Qionglai, China | 103/30 | 612 | 3 |
|  | ZH0125-S-10 | CQ1-1 | Chongqing, China | 106/29 | 287 | 2 |
|  | ZH0041-S-5 | XJ1-2 | Chengdu, China | 103/30 | 457 | 3 |
|  | ZH0160-S-2 | YN4 | Kunming, China | 102/24 | 1890 | 3 |
|  | ZH0168-S-3 | YN8-2 | Kunming, China | 102/24 | 1886 | 6 |
|  | ZH0171-S-3 | YN9-2 | Kunming, China | 102/24 | 1886 | 1 |
|  | ZH0062-S-5 | Y5-2 | Yaan,China | 103/30 | 607 | 2 |
|  | ZH0051-S-5 | XJ3 | Chengdu, China | 103/30 | 467 | 11 |
|  | ZH0104-S-7 | P2-1 | Chengdu, China | 104/30 | 432 | 5 |
|  | ZH0076-S-5 | Y11-2 | Yaan,China | 103/29 | 646 | 5 |
|  | ZH0173-S-3 | YN12 | Kunming, China | 102/24 | 1890 | 10 |
|  | ZH0159-S-2 | HB1 | Hubei, China | 115/30 | 690 | 8 |
|  | ZH0049-S-5 | XJ2-1 | Chengdu, China | 103/30 | 469 | 2 |
|  | ZH0088-S-6 | Q6-1 | Qionglai, China | 103/30 | 544 | 2 |
|  | ZH0108-S-8 | P3 | Chengdu, China | 103/30 | 624 | 2 |
|  | ZH0065-S-5 | Y6-2 | Yaan,China | 103/30 | 912 | 2 |
|  | ZH0034-S-0 | J1 | Jianyan, China |  |  | 2 |
|  | ZH0083-S-5 | L2'-2 | Leshan, China | 103/29 | 409 | 2 |
|  | ZH0079-S-5 | Y13 | Yaan,China | 103/29 | 646 | 2 |
|  | ZH0086-S-5 | L3-2 | Leshan, China | 103/29 | 353 | 2 |
|  |  | 7260 | Australia | --- | --- | 3 |
|  |  | 7760 | Australia | --- | --- | 6 |
|  |  | 9234 | South America | --- | --- | 7 |
|  |  | 9245 | Asia | --- | --- | 3 |
|  |  | 9264 | Pacific | --- | --- | 6 |
|  |  | 9595 | Jiten Khurana | --- | --- | 7 |
|  | ZH0055-S-5 | Y2-1 | Yaan,China | 103/30 | 576 | 3 |
|  | ZH0053-S-5 | Y1-1 | Yaan,China | 103/30 | 576 | 1 |
|  | ZH0143-S-12 | NJ3-2 | Neijiang, China | 119/25 | 7 | 8 |
|  | ZH0121-S-9 | KX2-1 | Chongqing, China | 108/30 | 192 | 5 |
|  | ZH0028-L-0 | V8 | Hanoi, Vietnam | 105/21 | 10 | 10 |
|  | ZH0017-L-0 | V9 | Hanoi, Vietnam | 105/21 | 10 | 8 |
|  | ZH0177-S-4 | HB2 | Wuhan, China | 114/29 | 643 | 2 |
|  | ZH0224-S-8 |  | Zigong, China | 104/24 | 521 | 2 |
|  | ZH0188-S-6 | 7776 | North |  |  | 7 |
|  | ZH0047-S-5 | Q5-2 | Qionglai, China | 103/30 | 698 | 2 |
|  | ZH0180-S-4 | CD3 | Chengdu, China | 104/30 | 436 | 2 |
|  |  |  |  |  |  |  |
| *Spirodel polyriza* | ZH0090-D-6 | JY3-1 | Jianyang, China | 104/30 | 430 | 1 |
|  | ZH0013-D-0 | V5-1 | Hu Zhiming, Vietnam | 106/10 | 5 | 4 |
|  | ZH0022-D-0 | V1-1 | --- | --- | --- | 1 |
|  | ZH0023-D-0 | V1-2 | Hanoi, Vietnam | 105/21 | 5 | 4 |
|  | ZH0043-D-5 | Q4-1 | Qionglai, China | 103/30 | 455 | 4 |
|  | ZH0094-D-7 | YN1-1 | Yuanyang, China | 102/23 | 312 | 6 |
|  | ZH0102-D-7 | GZ1-1 | Guangzhou, China | 113/23 | 13 | 5 |
|  | ZH0109-D-8 | P4-1 | Chengdu, China | 103/30 | 628 | 4 |
|  | ZH0132-D-10 | V10-1 | Hanoi, Vietnam | 105/21 | 10 | 4 |
|  | ZH0136-D-10 | V12-1 | Hanoi, Vietnam | 105/21 | 10 | 6 |
|  | ZH0167-D-3 | YN8-1 | Kunming, China | 102/24 | 1888 | 1 |
|  | ZH0006-D-0 | Q1 | Qionglai, China | 103/30 | 504 | 1 |
|  | ZH0003-D-0 | V7 | Hanoi, Vietnam | 106/21 | 59 | 3 |
|  | ZH0101-D-7 | SN1-3 | Shuining, China | 105/30 | 267 | 4 |
|  | ZH0067-D-5 | Y7-1 | Yaan, China | 13/30 | 902 | 5 |
|  | ZH0123-D-9 | KX1 | Chongqing, China | 108/30 | 192 | 5 |
|  | ZH0092-D-6 | WX1-1 | Wuxi, China | 120/30 | 11 | 5 |
|  | ZH0111-D-8 | Q7-1 | Qionglai, China | 103/30 | 546 | 2 |
|  | ZH0174-D-3 | YN11-1 | Kunming, China | 102/24 | 1890 | 8 |
|  | ZH0064-D-5 | Y6-1 | Yaan,China | 103/30 | 912 | 2 |
|  | ZH0082-D-5 | L2'-1 | Leshan, China | 103/29 | 409 | 2 |
|  | ZH0032-D-0 | V4-1 | Hanoi, Vietnam | 106/10 | 2 | 3 |
|  | ZH0099-D-7 | SN1-1 | Shuining, China | 105/30 | 267 | 2 |
|  | ZH0040-D-5 | XJ1-1 | Chengdu, China | 103/30 | 457 | 3 |
|  | ZH0085-D-5 | L3-1 | Leshan, China | 103/29 | 353 | 16 |
|  | ZH0008-D-0 | V1 | Hanoi, Vietnam | 105/21 | 9 | 9 |
|  | ZH0071-D-5 | Y9-1 | Yaan,China | 103/30 | 904 | 5 |
|  | ZH0046-D-5 | Q5-1 | Qionglai, China | 103/30 | 455 | 2 |
|  | ZH0192-D-6 | CQTN1 | Chongqing, China | 106/31 | 690 | 2 |
|  | ZH0203-D-7 | LS4 | --- | --- | --- | 2 |
|  | ZH0213-D-8 | NJ3-2 | Neijiang, China | 105/29 | 790 | 3 |
|  | ZH0228-D-8 | TJKYZ | --- | --- | --- | 3 |
|  |  | 9497 | Asia | --- | --- | 2 |
|  |  | 9500 | Europe | --- | --- | 2 |
|  |  | 9503 | Asia | --- | --- | 2 |
|  |  | 9504 | Asia | --- | --- | 2 |
|  |  | 9510 | Africa | --- | --- | 2 |
|  |  | 9256 | Europe | --- | --- | 2 |
|  |  | 9507 | Asia | --- | --- | 2 |
|  |  | 9609 | Bialowieza, Poland | --- | --- | 2 |
|  |  | 9610 | River Perebel, Russia | --- | --- | 2 |
|  |  | 9608 | Halina Gabrys, ?? | --- | --- | 2 |
|  |  | 9617 | Kerkini lake, Greece | --- | --- | 3 |
|  |  | 9618 | Trasimento lake, Perugia | --- | --- | 2 |
|  |  | 9612 | river Bug, Poland | --- | --- | 2 |
|  |  | 9603 | Lyon, France | --- | --- | 2 |
|  |  | 9560 | Szarvas, Hungary | --- | --- | 2 |
|  |  | 9620 | New Delhi, India | --- | --- | 12 |
|  |  | 9167 | South America | --- | --- | 13 |
|  |  | 9497 | Asia | --- | --- | 2 |
